# Bridging Parental Perceptions and Experiences of Family‐Centered Care in the Pediatric Intensive Care Unit: A Mixed‐Methods Study

**DOI:** 10.1111/jocn.70500

**Published:** 2026-08-11

**Authors:** Merve Azak, Naile Yörür, İrem Cafrı, Nihan Korkmaz

**Affiliations:** ^1^ Department of Pediatric Nursing, Florence Nightingale Faculty of Nursing Istanbul University‐Cerrahpasa Istanbul Türkiye; ^2^ Pediatric Intensive Care Unit Kocaeli City Hospital Kocaeli Türkiye; ^3^ Division of Pediatric Nursing, Department of Nursing, Faculty of Health Sciences Muğla Sıtkı Koçman University Muğla Türkiye

**Keywords:** family‐centered care, mixed methods study, parent participation, parental involvement, parents, pediatric intensive care unit, pediatric nursing

## Abstract

**Aim:**

To examine parents' perceptions and experiences of family‐centered care in a pediatric intensive care unit and explore how communication, participation in care, and contextual factors shape these experiences.

**Design:**

An explanatory sequential mixed‐methods design was used.

**Methods:**

In the quantitative phase, 50 parents of children hospitalized in the pediatric intensive care unit for at least 48 h completed the Descriptive Information Form and the Family‐Centered Care Assessment Scale. In the qualitative phase, 15 parents, selected through maximum variation sampling, participated in semi‐structured interviews. Quantitative data were analysed using descriptive and nonparametric methods, while qualitative data were analysed using reflexive thematic analysis. Findings from both phases were integrated during interpretation.

**Results:**

Parents generally reported positive perceptions of family‐centered care. Exploratory quantitative findings indicated higher family‐centered care and support scores among parents who reported greater participation in their child's care. Qualitative findings were organized into four themes: meanings and expectations of family‐centered care, communication and professional support, experiences of participation in care, and organizational and environmental influences on care. Integrated findings showed that respectful communication, emotional support, and meaningful parental involvement strengthened family‐centered care experiences. In contrast, inconsistent professional approaches, limited involvement in decision‐making, and inadequate physical conditions negatively influenced parental experiences.

**Conclusion:**

Parents' experiences of family‐centered care in the pediatric intensive care unit were shaped not only by communication with healthcare professionals but also by opportunities for participation, relational trust, and organisational conditions. Strengthening family‐centered care may require more consistent communication practices, greater parental involvement in care and decision‐making, and more family‐supportive care environments.

**Implications for the Profession and Patient Care:**

The findings highlight the importance of effective nurse‐parent communication, parental involvement in care, and supportive clinical environments in strengthening family‐centered care practices in pediatric intensive care units.

**Reporting Method:**

The study followed the Good Reporting of a Mixed Methods Study (GRAMMS) guidelines.

## Key Points

- What does this paper contribute to the wider global clinical community?
  ○ Family‐centered care in pediatric intensive care units is shaped not only by supportive communication but also by meaningful parental participation, relational trust, and organizational conditions.
  ○ Parents' involvement in care was influenced by nurses' communication styles, institutional routines, visitation policies, and the physical environment, highlighting important barriers and facilitators to implementing family‐centered care in intensive care settings.
  ○ Integrating quantitative and qualitative findings showed that supportive nurse‐parent communication alone may not ensure parents feel included in care and decision‐making processes, emphasizing the need for more participatory and family‐supportive clinical practices.

## 1 Introduction

Family‐centered care (FCC) is an approach that recognizes children and their families as essential partners in healthcare. Rather than viewing parents as visitors or passive recipients of information, FCC promotes respectful partnerships between families and healthcare professionals through information sharing, participation in care, shared decision‐making, and collaboration. These principles are considered fundamental to improving the health and well‐being of both children and their families and are widely accepted as the foundation of high‐quality pediatric care (Institute for Patient and Family‐Centered Care 2026). In pediatric intensive care units (PICUs), where children often require complex and highly specialized treatment, FCC has become an important component of care because parents are expected to remain involved in their child's care and decision‐making during one of the most stressful periods of hospitalization (Abukari and Schmollgruber 2023).

## 2 Background

Growing evidence indicates that FCC is associated with a range of positive outcomes for both children and their families in pediatric settings. Recent literature has reported that FCC interventions are associated with higher parental satisfaction, improved communication between families and healthcare professionals, greater parental involvement in care, and better preparedness for discharge (Aljawad et al. 2025; Tcharmtchi et al. 2024). Evidence from neonatal and pediatric settings suggests that family involvement may contribute to improvements in selected clinical and developmental outcomes. However, evidence regarding direct clinical outcomes among critically ill children in PICUs remains limited (Chen et al. 2025; Hodgson et al. 2024). In PICUs, meaningful parental involvement and shared decision‐making have been associated with greater trust in healthcare professionals, more positive care experiences, and stronger family‐professional partnerships (Azoulay et al. 2024; Charles et al. 2026).

Although FCC is widely accepted as the preferred model of pediatric care, implementing its principles in PICUs remains challenging. The technological nature of the PICU, the severity of children's conditions, infection prevention requirements, and the rapid pace of clinical decision‐making often limit opportunities for family involvement in care (Abukari and Schmollgruber 2023; Aljawad et al. 2025). These challenges affect not only the delivery of FCC but also parents' experiences during their child's hospitalization. Recent qualitative evidence shows that parents value clear communication, emotional support, and opportunities to participate in care, yet many continue to report difficulties in accessing information, uncertainty about their role, and inconsistent involvement in decision‐making (Dias Lessa et al. 2026). These experiences are particularly important because parents of critically ill children commonly report high levels of stress, anxiety, and psychological distress throughout the PICU admission (Andretta et al. 2025; Lisanti et al. 2024). This issue is particularly important because the Post‐Intensive Care Syndrome–pediatrics (PICS‐p) framework recognizes family engagement as a key factor influencing the long‐term well‐being of both critically ill children and their families following PICU admission (Curley et al. 2024).

Parents consistently identify communication, emotional support, and opportunities to participate in care as central elements of a positive PICU experience (Dias Lessa et al. 2026), and FCC interventions that strengthen these aspects have been associated with greater parental trust and more positive care experiences (Tcharmtchi et al. 2024).

Despite the growing body of research on FCC in pediatric intensive care, important gaps remain. Existing research has predominantly examined parents' perceptions using standardized questionnaires or explored their experiences through qualitative methods. While this body of research has generated valuable evidence, recent reviews also highlight considerable variation in FCC interventions and outcome measures across PICUs, limiting comparisons between studies and hindering a comprehensive understanding of FCC implementation (Charles et al. 2026). In addition, a qualitative systematic review concluded that parents' experiences of FCC in the PICU remain insufficiently synthesized and emphasized the need for further research to better understand how parents experience participation in care (Dias Lessa et al. 2026). A mixed‐methods approach is well suited to address these gaps by integrating quantitative assessments with parents' narratives, offering deeper insight into how FCC is experienced in clinical practice (Creswell and Plano Clark 2018; Fetters et al. 2013). Therefore, this mixed‐methods study aimed to evaluate parents' perceptions and experiences of FCC in a PICU by integrating quantitative and qualitative findings. By doing so, the study may also provide context‐specific evidence from a Turkish PICU, a healthcare setting that remains underrepresented in the international FCC literature.

## 3 The Study

### 3.1 Aim

To quantitatively evaluate parents' perceptions of FCC in the PICU and identify factors influencing these perceptions. In addition, qualitative findings were used to provide an in‐depth understanding of parental experiences and contextual factors shaping FCC.

### 3.2 Research Question

What are the perceptions and experiences of parents of children receiving treatment in the PICU regarding FCC?

### 3.3 Quantitative Sub‐Questions

– What are the levels of parents' perceptions of FCC?
– Do these perceptions differ according to sociodemographic characteristics and care process variables?

### 3.4 Qualitative Sub‐Questions

– How do parents define FCC?
– What are their experiences regarding FCC?
– What difficulties do they encounter in participating in the care process?
– What factors facilitate and hinder FCC?

## 4 Methods

### 4.1 Design

This study was conducted using an explanatory sequential mixed‐methods design to examine the perceptions and experiences of parents of children receiving treatment in the PICU regarding FCC (Creswell and Plano Clark 2018). Within this design, the study was carried out in two sequential phases. In the first phase, parents' perceptions of FCC were quantitatively assessed; in the second phase, qualitative data were collected and analysed to explain, elaborate on, and provide contextual understanding of the quantitative findings.

The study was guided by a pragmatic paradigm, which allows for a holistic evaluation of the complex and context‐dependent nature of FCC. While the quantitative component revealed general patterns in parents' perceptions of FCC, the qualitative component enabled an understanding of the communicational, relational, and organizational experiences underlying these perceptions. Thus, the study aimed to reveal not only parents' levels of perception regarding FCC but also the experiential processes shaping these perceptions.

In line with the mixed‐methods approach, integration occurred at the stages of study design, sample selection, data analysis, and interpretation. The qualitative phase was informed by the quantitative findings and aimed to explain convergent, divergent, and contextual patterns emerging from the quantitative results (Fetters et al. 2013).

The study was reported in accordance with the Good Reporting of a Mixed Methods Study (GRAMMS) checklist for mixed‐methods research (O'Cathain et al. 2008), the Strengthening the Reporting of Observational Studies in Epidemiology (STROBE) checklist for the quantitative component (von Elm et al. 2008), and the Consolidated Criteria for Reporting Qualitative Research (COREQ) checklist for the qualitative component (Tong et al. 2007).

### 4.2 Study Setting and Sampling

The study was conducted in the PICU of a city hospital in Türkiye between September 2025 and February 2026. The study population consisted of parents of children who were hospitalized in the PICU for longer than 48 h during the study period. The study was conducted in two sequential phases: quantitative and qualitative.

In the quantitative phase, the inclusion criteria were defined as follows: (1) being 18 years of age or older, (2) being able to communicate in Turkish, and (3) volunteering to participate in the study. Parents whose child's clinical condition was critically unstable, who were experiencing physical or psychological distress severe enough to impair communication, and who were considered unsuitable for interview by the intensive care team during the data collection process were excluded from the study. A minimum hospitalization period of 48 h was established to ensure that parents had sufficient experience with the intensive care environment, communication with healthcare professionals, and care processes. The quantitative phase was primarily designed to describe parents' perceptions of FCC and to identify exploratory patterns that could inform the subsequent qualitative phase. The primary quantitative outcome was the total score of the Family‐Centered Care Assessment Scale. Because the number of eligible parents during the study period was limited, the sample size was calculated using a finite population approach. Based on hospital records from the same six‐month period in the previous year, 58 parents were estimated to meet the eligibility criteria. Using OpenEpi with a 95% confidence level and a 5% margin of error, the minimum required sample size was determined to be 50 parents. During the data collection period, all eligible parents were consecutively invited to participate, and 50 parents completed the quantitative phase. No a priori power analysis was conducted to detect between‐group differences in FCCAS scores because the quantitative phase was not designed as a hypothesis‐testing study. Therefore, inferential analyses were considered exploratory and interpreted cautiously.

In the qualitative phase, in accordance with the explanatory sequential mixed‐methods design, participants were purposefully selected from among the parents included in the quantitative phase using maximum variation sampling. The sampling strategy aimed to capture diverse FCC experiences and to provide an in‐depth explanation of convergent and divergent quantitative findings. Participant selection considered variation in parental characteristics, including educational level, employment status, number of children, perceived participation in care, communication experiences with healthcare professionals, and perceived social support, as well as child‐related clinical characteristics such as diagnosis group, presence of chronic illness, previous hospitalization or PICU admission, and length of PICU stay. Parents with differing levels of FCC perceptions identified during the quantitative phase were intentionally included to ensure variation in both positive and challenging care experiences.

Participant recruitment continued until data saturation was achieved. Preliminary coding was conducted after each interview, allowing data collection and analysis to proceed concurrently. Data saturation was assessed by the researcher (IC), who conducted the interviews and performed the preliminary coding, and was confirmed through regular discussions with the research team. Saturation was considered to have been reached when no new codes, categories, or themes emerged across successive interviews. After the 13th interview, no new codes or themes were identified. Two additional interviews were subsequently conducted to confirm that no new conceptual insights emerged, resulting in a final qualitative sample of 15 parents.

### 4.3 Instruments

#### 4.3.1 Demographic Information Form

This form, developed by the researchers in line with the literature, was used to assess the sociodemographic characteristics of the parents and the clinical characteristics of the children (Stalder et al. 2024; Terp et al. 2021). The form included questions regarding parents' age, sex, educational status, employment status, number of children, perceived level of social support, perceptions of communication with healthcare professionals, and participation in the care process. In addition, clinical characteristics of the children, including age, sex, presence of chronic illness, diagnosis group, and length of stay in the intensive care unit, were recorded.

#### 4.3.2 Family‐Centered Care Assessment Scale (FCCAS)

The FCCAS, developed by Tas Arslan et al. (2019), assesses parents' perceptions of FCC and enables parents of hospitalized children to evaluate FCC practices. It is a valid and reliable instrument for measuring FCC from the parental perspective in pediatric clinical settings. The scale development study was conducted with parents of children hospitalized in the pediatric clinics of two university hospitals in Türkiye, and the psychometric evaluation was performed with 360 parents. The FCCAS consists of 21 items and three subdimensions: support (10 items), collaboration (8 items), and respect (3 items). The support subdimension evaluates the emotional and informational support provided by healthcare professionals to parents; the collaboration subdimension evaluates parental involvement in the care process and partnership with healthcare professionals; and the respect subdimension evaluates respect for parents' opinions, preferences, and individual characteristics. The scale is structured as a 5‐point Likert‐type instrument (1 = strongly disagree, 5 = strongly agree). The lowest and highest scores that could be obtained from the scale are 21 and 105. Higher scores on the scale indicate more positive parental evaluations of FCC practices. In the original study, the Cronbach's alpha coefficient was reported as 0.94 for the total scale. Cronbach's alpha values for the subdimensions were reported as 0.89 for support, 0.90 for collaboration, and 0.82 for respect. In addition, the authors reported a Pearson test–retest correlation of *r* = 0.90 (*p* < 0.001); however, an intraclass correlation coefficient (ICC) was not reported. In the present study, the Cronbach's alpha coefficient of the total scale was 0.87. The Cronbach's alpha values for the subscales were 0.80 for support, 0.79 for collaboration, and 0.55 for respect. While the total scale and the support and collaboration subscales demonstrated acceptable‐to‐good internal consistency, the reliability of the respect subscale was relatively low.

#### 4.3.3 Semi‐Structured Interview Guide

A semi‐structured interview guide developed by the researchers in accordance with the literature was used to collect qualitative data (Jarvis et al. 2024; Stalder et al. 2024; Terp et al. 2021). The interview guide was structured to cover four main areas: (1) how FCC was defined by parents, (2) parental experiences during the intensive care process, (3) experiences related to participation in the care process and communication with healthcare professionals, and (4) factors facilitating and hindering FCC. The interview guide consisted of open‐ended questions and was conducted using a flexible structure that allowed participants to express their experiences in detail.

### 4.4 Data Collection

The data collection process was conducted in two sequential phases in accordance with the explanatory sequential mixed‐methods design. Quantitative data were collected between September and December 2025 using face‐to‐face interviews. Data collection was conducted during time periods that would not disrupt the parents' clinical care process, and each application lasted approximately 15 min. The data collection forms were reviewed after administration to minimize missing data. All participants were informed about the purpose of the study, and it was emphasized that participation was voluntary.

Following the completion of the preliminary analysis of the quantitative data, the qualitative data collection process was initiated between January and February 2026. Semi‐structured interviews were conducted face‐to‐face in a quiet environment that ensured privacy and was not affected by clinical workflow. The interviews lasted approximately 30–45 min, and interviews were audio‐recorded with the written consent of the participants.

All interviews were conducted by the same researcher, who had received training in qualitative research methods, had 2 years of clinical experience in a pediatric intensive care unit, and had been working as a research assistant in pediatric nursing for the past 2 years while pursuing doctoral‐level education. The researcher's previous clinical experience facilitated rapport with participants and supported a context‐sensitive understanding of their experiences. At the same time, the researchers acknowledged that this background could influence data collection and interpretation. To minimize potential bias, the interviewer used open‐ended questions, avoided leading participants during interviews, maintained reflexive field notes throughout data collection and analysis, and regularly discussed emerging codes and themes with the research team to challenge assumptions and enhance analytical rigour.

In the qualitative phase, the processes of data collection and preliminary analysis were conducted iteratively. Following each interview, preliminary coding was performed to evaluate whether new codes, categories, or themes had emerged. Data saturation was considered achieved at the point when no new meaningful codes or themes emerged.

### 4.5 Data Analysis

Quantitative data were analysed using IBM SPSS Statistics for Windows, Version 23.0 (IBM Corp., Armonk, NY, USA). Mean, standard deviation, median, first and third quartile values (Q1–Q3), frequency, and percentage values were calculated to describe participants' sociodemographic and clinical characteristics and scale scores. The normality of the data distribution was evaluated using the Shapiro–Wilk test. Scale scores that did not meet the normality assumption were summarized using median and Q1–Q3 values. In group comparisons, the Mann–Whitney *U* test was used for comparisons between two independent groups, and the Kruskal–Wallis test was used for comparisons among three or more groups, according to data distribution and group characteristics. For variables including three groups, the significance level after Bonferroni correction was accepted as *p* < 0.017. For variables in which a significant difference was identified with the Kruskal–Wallis test, pairwise comparisons were performed using the Bonferroni‐corrected Mann–Whitney *U* test. For the Mann–Whitney *U* test, the effect size was calculated as the standardized rank‐based effect size *r* using the formula *r* = |*Z*|/√*N*, where *Z* is the standardized test statistic and *N* is the total number of observations. Effect sizes were interpreted as 0.10 for a small effect, 0.30 for a medium effect, and 0.50 for a large effect (Fritz et al. 2012). For the Kruskal–Wallis test, effect size was calculated using the epsilon‐squared (*ε* ^2^) value. In all analyses, the overall significance level was accepted as *p* < 0.05. Because multiple subgroup comparisons were conducted across the FCCAS total and subscale scores, these analyses were considered exploratory. Apart from Bonferroni correction for post hoc pairwise comparisons following significant Kruskal–Wallis tests, no additional correction was applied across all subgroup analyses; therefore, statistically significant findings were interpreted cautiously. Accordingly, inferential analyses were interpreted as exploratory and were primarily used to identify quantitative patterns that informed the subsequent qualitative phase and data integration.

Qualitative data were analysed using the reflexive thematic analysis approach described by Braun and Clarke (2006). The analysis process was conducted in line with an inductive approach, focusing on allowing themes to emerge from the data rather than determining them in advance. All interviews were transcribed verbatim and compared with the audio recordings to ensure accuracy.

The researchers first read the transcripts repeatedly to achieve familiarity with the data. Subsequently, open coding was performed, and codes that were conceptually related were grouped together to form subthemes. The subthemes were then organized under broader main themes according to higher‐level conceptual patterns. The themes were repeatedly reviewed and refined by the research team, and consensus was reached regarding the final thematic structure.

To enhance the trustworthiness of the qualitative findings, the criteria of credibility, dependability, confirmability, and transferability were taken into consideration. In this context, independent coding was conducted, the analysis process was systematically documented, reflexive notes were maintained, and the consistency of interpretations was evaluated through discussions among the researchers. To support transferability, participant characteristics, the data collection process, and the analysis method were reported in detail. Direct quotations from participant statements were used to support the themes.

In line with the mixed‐methods approach, data integration was conducted during the interpretation phase. While the quantitative findings revealed parents' overall levels of perception regarding FCC and the associated factors, the qualitative findings contributed to explaining the experiential and contextual processes underlying these patterns. Narrative comparison and a joint display approach were used during the integration process. Quantitative findings were compared with related qualitative themes to identify convergent, complementary, and divergent patterns across the two datasets. In particular, quantitative relationships related to participation in the care process, communication with healthcare professionals, perceptions of support, and physical environmental conditions were interpreted together with parental perspectives. This approach strengthened the clinical significance of the findings by enabling statistical trends to be explained holistically through parental experiences.

### 4.6 Ethical Considerations

This study was conducted in accordance with the principles of the Declaration of Helsinki. Ethical approval was obtained from the University Ethics Committee (2025/427), and institutional permission was granted by the participating hospital. Written informed consent was obtained from all participants prior to data collection. Participation was voluntary, confidentiality was assured, and participants were informed of their right to withdraw from the study at any time without penalty.

## 5 Findings

### 5.1 Quantitative Findings

In the quantitative phase, the mean age of the parents was 34.7 ± 4.4 years, and most participants were female (*n* = 46, 92%), mothers (*n* = 46, 92%), and married (*n* = 50, 100%). High school education was the predominant educational category (*n* = 20, 40%), and most parents were unemployed (*n* = 37, 74%). Most families had two children (*n* = 32, 64%). Health literacy was most frequently reported at a moderate level (*n* = 22, 44%), while the vast majority of parents reported receiving social support during the care process (*n* = 46, 92%). Perceptions of communication with healthcare professionals were mostly moderate (*n* = 24, 48%) and high (*n* = 21, 42%). Participation in the care process was evaluated as high or very high by most parents (*n* = 33, 66.0%), with very high being the most common single category (*n* = 24, 48.0%) (Table 1).

**TABLE 1:** Descriptive characteristics and care‐related experiences of parents.

Variables	Quantitative phase (*n* = 50)	Qualitative phase (*n* = 15)
	Mean ± SD	Mean ± SD
Age (years)	34.7 ± 4.4	35.7 ± 5.0
	*n* (%)	*n* (%)
Sex		
Female	46 (92)	13 (86.7)
Male	4 (8)	2 (13.3)
Relationship to child		
Mother	46 (92)	13 (86.7)
Father	4 (8)	2 (13.3)
Marital status		
Married	50 (100)	15 (100)
Single	—	—
Divorced	—	—
Widowed	—	—
Educational status		
Primary/middle school	19 (38)	8 (53)
High school	20 (40)	1 (6.7)
University and more	11 (22)	6 (40)
Employment status		
Employed	13 (26)	2 (13.3)
Unemployed	37 (74)	13 (86.7)
Family type		
Nuclear	49 (98)	14 (93.3)
Divided	1 (2)	1 (6.7)
Total number of children		
One	7 (14)	3 (20)
Two	32 (64)	9 (60)
Three and more	11 (22)	3 (20)
Awareness of the concept of FCC		
Yes	—	—
No	50 (100)	15 (100)
Health literacy level (understanding health information)		
Very low	5 (10)	1 (6.7)
Low	13 (26)	6 (40)
Moderate	22 (44)	6 (40)
High	10 (20)	2 (13.3)
Very high	—	—
Receiving social support during the care process		
Yes	46 (92)	14 (93.3)
No	4 (8)	1 (6.7)
Perception of communication with healthcare professionals		
Moderate	24 (48)	6 (40)
High	21 (42)	8 (53.3)
Very high	5 (10)	1 (6.7)
Level of participation in the care process		
None	2 (4)	1 (6.7)
Low	1 (2)	—
Moderate	14 (28)	4 (26.7)
High	9 (18)	2 (13.3)
Very high	24 (48)	8 (53.3)

The mean age of the parents included in the qualitative phase was 35.7 ± 5.0 years, and most participants were female (*n* = 13, 86.7%), mothers (*n* = 13, 86.7%), and married (*n* = 15, 100%). The most common educational level was primary/middle school (*n* = 8, 53.3%), and the majority of parents were unemployed (*n* = 13, 86.7%). Most participants had two children (*n* = 9, 60%). Health literacy levels were predominantly reported as low (*n* = 6, 40%) and moderate (*n* = 6, 40%). The vast majority of parents stated that they received social support during the care process (*n* = 14, 93.3%). Perceptions of communication with healthcare professionals were mostly high (*n* = 8, 53.3%) and moderate (*n* = 6, 40%). Participation in the care process was evaluated as high or very high by most parents in the qualitative phase (*n* = 10, 66.7%), with very high being the most common single category (*n* = 8, 53.3%) (Table 1).

In the quantitative phase, the mean age of the children was 71.9 ± 53.2 months (range: 2–204 months), and most were female (*n* = 30, 60%). The most common current disease group was neurological disorders (*n* = 18, 36%), followed by respiratory diseases (*n* = 15, 30%), cardiovascular diseases (*n* = 11, 22%), and infections (*n* = 6, 12%). Some of the children had an underlying chronic illness (*n* = 11, 22%). Previous hospitalization history was reported in 36% of children. A history of previous intensive care admission was reported less frequently (*n* = 6, 12%). Most children in the quantitative phase had a PICU stay of 3–14 days (*n* = 41, 82%), with the most common category being 3–7 days (*n* = 25, 50%) (Table 2).

**TABLE 2:** Descriptive and clinical characteristics of children.

	Quantitative phase (*n* = 50)	Qualitative phase (*n* = 15)
	Mean ± SD	Mean ± SD
Child's age (months)	71.9 ± 53.2	75.6 ± 57.3
	*n* (%)	*n* (%)
Sex		
Female	30 (60)	10 (66.7)
Male	20 (40)	5 (33.3)
Current disease group		
Respiratory disease	15 (30)	8 (53.3)
Neurological disorder	18 (36)	4 (26.7)
Cardiovascular disease	11 (22)	2 (13.3)
Infection	6 (12)	1 (6.7)
Presence of underlying chronic illness		
Yes	11 (22)	5 (33.3)
No	39 (78)	10 (66.7)
History of previous hospitalization		
Yes	18 (36)	4 (26.7)
No	32 (64)	11 (73.3)
History of previous intensive care admission		
Yes	6 (12)	2 (13.3)
No	44 (88)	13 (86.7)
Length of stay in the PICU		
3–7 days	25 (50)	8 (53.3)
8–14 days	16 (32)	5 (33.3)
15–30 days	4 (8)	—
≥ 31 days	5 (10)	2 (13.3)

*Note:* Length of PICU stay was collected and reported as an ordinal categorical variable.

In the qualitative phase, the mean age of the children was 75.6 ± 57.3 months, and most were female (*n* = 10, 66.7%). The most common current disease group was respiratory diseases (*n* = 8, 53.3%), followed by neurological disorders (*n* = 4, 26.7%), cardiovascular diseases (*n* = 2, 13.3%), and infections (*n* = 1, 6.7%). Some of the children had an underlying chronic illness (*n* = 5, 33.3%). While a history of previous hospitalization was present in a proportion of the children (*n* = 4, 26.7%), a history of previous intensive care admission was reported less frequently (*n* = 2, 13.3%). Similarly, most children in the qualitative phase had a PICU stay of 3–14 days (*n* = 13, 86.7%), with the most common category being 3–7 days (*n* = 8, 53.3%) (Table 2).

The median total FCCAS score was 81.0 (Q1–Q3: 73.0–96.3). Descriptive statistics for the total scale and subscale scores are presented in Table 3.

**TABLE 3:** Descriptive statistics of the Family‐Centered Care Assessment Scale total and subscale scores.

Scale/subdimension	Median	(Q1–Q3)
Total Score	81.0	(73.0–96.3)
Support subdimension	38.0	(33.0–46.0)
Collaboration subdimension	31.5	(28.8–37.5)
Respect subdimension	14.0	(12.0–15.0)

According to employment status, a significant difference was identified only in the respect subdimension; the median score was 15.0 (13.5–15.0) for employed parents and 13.0 (12.0–15.0) for unemployed parents (*U* = 151.00; *p* = 0.039; *r* = 0.28). According to the total number of children, the total score and support subdimension differed significantly; these scores were higher among parents with one child, and Bonferroni‐corrected post hoc analysis showed that the difference was between the groups with one child and three or more children [total score: *χ* ^2^ = 8.00; *p* = 0.018; *ε* ^2^ = 0.13; post hoc *p* = 0.004, *r* = 0.67; support: *χ* ^2^ = 8.14; *p* = 0.017; *ε* ^2^ = 0.13; post hoc *p* = 0.007, *r* = 0.64]. In addition, parents reporting a high level of participation in the care process had higher total scores and support subdimension scores (*U* = 153.50; *p* = 0.009; *r* = 0.37 and *U* = 165.00; *p* = 0.018; *r* = 0.34, respectively). No significant differences were found in comparisons according to educational status, health literacy, perception of communication, length of stay in the intensive care unit, and other subdimension comparisons that did not demonstrate significance (*p* > 0.05) (Table 4).

**TABLE 4:** Comparison of Family‐Centered Care Assessment Scale total and subscale scores according to parental characteristics and care‐related variables.

Variables	*n*	Total median (Q1–Q3)	Support median (Q1–Q3)	Collaboration median (Q1–Q3)	Respect median (Q1–Q3)
Educational status					
Primary/middle school	19	79.0 (69.0–97.0)	38.0 (33.0–47.0)	29.0 (25.0–39.0)	14.0 (12.0–15.0)
High school	20	83.5 (77.0–93.3)	38.5 (33.8–44.3)	33.5 (29.3–36.8)	14.0 (12.0–15.0)
University and more	11	77.0 (69.0–101.0)	37.0 (32.0–46.0)	30.0 (26.0–40.0)	14.0 (12.0–15.0)
Test/*p*		*χ* ^2^ = 1.439; *p* = 0.487	*χ* ^2^ = 0.219; *p* = 0.960	*χ* ^2^ = 3.179; *p* = 0.204	*χ* ^2^ = 0.089; *p* = 0.957
Employment status					
Employed	13	84.0 (76.5–98.0)	39.0 (34.5–45.5)	36.0 (28.0–39.0)	15.0 (13.5–15.0)
Unemployed	37	79.0 (73.0–96.5)	37.0 (33.0–46.5)	31.0 (28.5–36.5)	13.0 (12.0–15.0)
Test/*p*		*U* = 205.00; *p* = 0.432	*U* = 224.00; *p* = 0.715	*U* = 205.00; *p* = 0.430	*U* = 151.00; *p* = 0.039*; *r* = 0.28
Total number of children					
One^a^	7	99.0 (90.0–102.0)	46.0 (42.0–49.0)	39.0 (29.0–40.0)	14.0 (12.0–15.0)
Two^b^	32	79.5 (73.3–95.5)	38.0 (33.3–44.3)	30.0 (28.0–37.0)	14.0 (12.0–15.0)
Three and more^c^	11	74.0 (67.0–84.0)	33.0 (30.0–39.0)	31.0 (26.0–33.0)	14.0 (12.0–15.0)
Test/*p*		*χ* ^2^ = 8.00; *p* = 0.018*	*χ* ^2^ = 8.14; *p* = 0.017*	*χ* ^2^ = 5.64; *p* = 0.060	*χ* ^2^ = 0.53; *p* = 0.767
		*ε* ^2^ = 0.13	*ε* ^2^ = 0.13		
		a > c (*p* = 0.004** *r* = 0.67)	a > c (*p* = 0.007** *r* = 0.64)		
Health literacy level					
Low	18	79.0 (73.0–94.5)	37.5 (33.8–42.8)	30.5 (27.8–37.5)	13.5 (12.0–15.0)
Moderate/high	32	84.0 (73.3–97.8)	38.5 (32.3–47.0)	33.0 (29.0–38.5)	14.0 (12.0–15.0)
Test/*p*		*U* = 260.50; *p* = 0.578	*U* = 276.00; *p* = 0.808	*U* = 256.00; *p* = 0.516	*U* = 259.00; *p* = 0.541
Perception of communication					
Moderate	24	76.5 (73.0–87.8)	36.5 (32.3–39.8)	29.5 (27.3–36.0)	13.0 (12.0–15.0)
High/very high	26	86.5 (77.0–99.3)	40.0 (35.5–47.3)	33.5 (29.0–39.0)	14.0 (12.0–15.0)
Test/*p*		*U* = 220.50; *p* = 0.075	*U* = 230.50; *p* = 0.113	*U* = 220.00; *p* = 0.073	*U* = 275.00; *p* = 0.454
Level of participation in the care process					
Low/moderate	17	73.0 (67.0–87.0)	33.0 (28.0–40.5)	29.0 (25.5–34.0)	12.0 (12.0–14.5)
High	33	84.0 (77.0–99.0)	39.0 (36.0–47.0)	34.0 (29.0–39.0)	14.0 (12.0–15.0)
Test/*p*		*U* = 153.50; *p* = 0.009*; *r* = 0.370	*U* = 165.00; *p* = 0.018*; *r* = 0.340	*U* = 186.50; *p* = 0.053	*U* = 194.00; *p* = 0.065
Length of stay in the PICU					
3–7 days	25	84.0 (77.0–95.0)	38.0 (34.0–43.5)	32.0 (29.0–38.0)	14.0 (12.0–15.0)
≥ 8 days	25	77.0 (71.0–99.0)	37.0 (33.0–47.0)	31.0 (26.5–37.5)	14.0 (12.0–15.0)
Test/*p*		*U* = 283.50; *p* = 0.573	*U* = 302.00; *p* = 0.838	*U* = 268.50; *p* = 0.391	*U* = 292.00; *p* = 0.679

*Note:* Values are presented as median (Q1–Q3). *p < 0.05; **p < 0.017 after Bonferroni correction. *χ* ^2^: Kruskal–Wallis test; *U*: Mann–Whitney *U* test; *r*: standardized rank‐based effect size for the Mann–Whitney *U* test; *ε* ^2^: effect size for the Kruskal–Wallis test. Different superscript letters (a–c) indicate significant differences between groups.

### 5.2 Qualitative Findings

Following analysis of the qualitative data, four main themes were identified that explained parents' experiences of FCC: (1) parental perceptions and expectations regarding FCC, (2) communication with nurses and perceived support, (3) experiences of involvement in the child's care, and (4) factors facilitating and hindering FCC.

#### 5.2.1 Parental Perceptions and Expectations Regarding Family‐Centered Care

During the interviews, parents described FCC as a concept closely associated with being present with their child, participating in care processes, and being recognized as an important part of care. Although participants differed in how they described FCC, their definitions reflected interconnected experiences within the PICU. Rather than providing direct conceptual definitions, parents interpreted FCC through their own experiences in the PICU.

Many parents emphasized that physically being with their child and participating in caregiving activities were essential components of FCC. For these parents, involvement in daily caregiving activities contributed not only to maintaining emotional closeness with their child but also to preserving continuity in their parental role throughout hospitalization. One participant described FCC as follows:

I think FCC means that my child receives good care and that I can also provide some of that care while we are in the intensive care unit. (Participant 8)

Some parents emphasized the importance of having their knowledge and opinions acknowledged by healthcare professionals. One father described FCC as a collaborative care process between parents and healthcare professionals:

I am my child's father. I am one of the two people who know my child best. For that reason, my opinion should be considered regarding every procedure performed for my child. To me, that is what FCC means. (Participant 7)

Parents' perceptions of FCC were also closely related to their expectations of nurses. Across many interviews, parents emphasized that their greatest expectation was for their child to receive attentive and high‐quality care. Feeling reassured about their child's health reduced parental stress throughout hospitalization. Educational support provided by nurses was therefore associated with preparation for post‐discharge caregiving and increased confidence. One participant explained the importance of receiving education and support from nurses as follows:

My biggest fear was how I would take care of my child at home. My child was receiving excellent care in the hospital, but I kept worrying about whether I would provide poor care at home and whether my child would end up back in the hospital because of me. That is why I hoped to receive good education during hospitalization. It happened exactly as I hoped. They provided us with excellent education. As my child was getting close to discharge, they explained all the care needs and showed me how to manage everything. (Participant 3)

Parents of children with chronic illnesses often compared hospital care with their caregiving routines at home and emphasized the importance of care quality during hospitalization. These parents viewed themselves not only as caregivers but also as individuals with extensive knowledge and long‐term experience related to their child's condition. One participant expressed this expectation as follows:

I have been taking care of my child myself for 16 years. But sometimes when we are hospitalized, my child ends up looking as though nobody has cared for them at all. At home my child never develops pressure injuries, but this has happened several times in the hospital. That is why my biggest expectation is for my child to receive even better care than at home and to be discharged in a cleaner and healthier condition. (Participant 4)

#### 5.2.2 Communication With Nurses and Perceived Support

Parents' experiences related to FCC were strongly associated with their communication with nurses and the emotional support they received throughout their child's stay in the PICU. Across interviews, participants frequently associated supportive communication with reduced anxiety and increased trust. For many parents, nurses' attitudes and communication styles influenced not only their perceptions of care quality but also their willingness to participate in care processes.

Most parents described positive communication experiences with nurses and emphasized the importance of compassionate and supportive interactions during this highly stressful period. Observing nurses communicate warmly and attentively with their children strengthened parents' trust in the healthcare team. One participant described this experience as follows:

The nurses were so kind that they treated my child as if they were caring for their own child, which made me feel very reassured. Seeing that my child did not cry when they were with the nurses comforted me a lot. When I knew that a nurse I trusted would be taking care of my child, I could sleep peacefully at home that night. (Participant 3)

Similarly, many parents emphasized the importance of supportive communication directed toward the family. Participants reported that positive communication with nurses facilitated learning, reduced stress, and increased their confidence in providing care. One participant described this experience as follows:

The nurses cared very much about our children. But I think the way they communicated with us was equally important. For example, during education sessions, they treated me so well that I was able to learn everything easily and without stress. They supported me every time I came to the hospital. (Participant 5)

In contrast, some participants also described negative communication experiences and perceived insufficient support. These experiences adversely affected parents' emotional well‐being. One participant expressed concern that some nurses did not demonstrate the level of sensitivity expected in the PICU setting:

I think some nurses do not show the extra sensitivity that pediatric intensive care requires. Children should not be treated the same way adults are treated, and the same applies to families. Sometimes they create a negative atmosphere even through their facial expressions, without saying anything. (Participant 12)

In some cases, negative communication experiences also influenced parents' willingness to participate in their child's care. Parents described feeling uncertain about whether their involvement in care would be welcomed, supported, or encouraged by healthcare professionals. One participant explained this hesitation as follows:

Forget asking for information—there were nurses I felt uncomfortable even asking if I could clean my child's face or touch my child's hand. I knew they would not support me. Because of that, there were things I wanted to do for my child but could not bring myself to do. (Participant 14)

#### 5.2.3 Experiences of Involvement in the Child's Care

Nearly all parents who participated in the interviews reported being involved in their child's care during the PICU stay. However, parents' narratives suggested that this involvement was not consistently perceived as part of the FCC process. Across interviews, parents emphasized that nurses' attitudes, communication styles, and willingness to encourage participation played an important role in shaping their experiences of involvement in care.

Many parents indicated that parental involvement in care was generally influenced more by encouragement from nurses than by institutional routines. Although parents expressed willingness to participate in care, they emphasized expecting support from healthcare professionals before becoming actively involved. Such support helped reduce parents' hesitations regarding caregiving activities and alleviated stress related to the post‐discharge period. One participant explained how even a small opportunity to participate in care during hospitalization increased both confidence and hope:

Even though I had entered the intensive care unit many times before, no nurse had ever involved me in providing care. One day, a nurse allowed me to clean my daughter's face and mouth. After that day, I started participating in her care every day. It made me feel excited as well. I began to feel that we might finally be able to go home. (Participant 9)

For parents of children with chronic illnesses, participation in care was described as beginning earlier and occurring more rapidly during hospitalization. These parents reported that nurses tended to collaborate more closely with them from the beginning of the PICU admission. They emphasized that mutual trust and prior caregiving experience facilitated a more collaborative care relationship. One participant described this experience as follows:

When we were first admitted, I think the nurses realized that I knew what I was doing when I told them I had been caring for my child for 16 years. I was not staying in the intensive care unit all the time, but every day when I came in, we provided care together. The nurses trusted me in that regard. And I listened to them to learn what new things I could learn from them. (Participant 4)

Similarly, one parent emphasized the importance of parental knowledge and active involvement in care‐related decisions. This participant explained feeling excluded from routine caregiving practices initially but later became more involved after communicating with nurses:

I wanted to provide my child's care myself. There were actually two reasons for this. First, I did not want a male nurse providing my child's hygiene care. Second, no one can care for my child as well as I can. I am the person who knows best what my child likes and what my child needs. At first, the nurses carried out their routine practices, but they never asked for my opinion or support. Later, I spoke with them, and I started doing things in ways that suited my child and made my child comfortable. (Participant 7)

Parents also reported that their child's clinical condition influenced their willingness to participate in care. Many participants stated that they had not considered becoming involved in caregiving activities while their child was intubated. During these periods, parents expressed fear of harming their child and believed it was more appropriate for nurses to provide care. One participant described this hesitation as follows:

I did not provide care while my child was intubated, and honestly, I do not think I would have wanted to. I would have been too afraid of doing something wrong. Because my child's condition was critical at that time, I think it was more appropriate for the nurses to provide all the care. (Participant 14)

#### 5.2.4 Factors Facilitating and Hindering Family‐Centered Care

Parents described various factors that either facilitated or hindered the implementation of FCC within the PICU setting. While supportive communication, collaboration with families, and improvements in the child's clinical condition facilitated parental involvement, participants also emphasized that institutional routines, physical environmental conditions, and limitations related to access to information created barriers to FCC. Although both parents and healthcare professionals appeared to value FCC, its implementation was frequently limited by organizational and structural constraints.

Parents reported that participation in their child's care became easier as their child's clinical condition improved and the likelihood of discharge increased. This process enhanced parents' self‐confidence and willingness to engage in caregiving activities. In addition, nurses' supportive and encouraging attitudes played a facilitating role by helping parents feel more comfortable participating in the care process.

Despite these positive experiences, parents frequently reported that the physical environment of the PICU was insufficient to support family presence and participation in care. Participants particularly emphasized the lack of family‐oriented facilities, such as dedicated sinks and appropriate resting areas. One participant questioned the absence of family‐specific hygiene facilities within the PICU setting as follows:

Because of infection precautions, families are allowed into the intensive care unit only once a day. But when we stay in the ICU, we constantly have to leave because there is no sink available. Doesn't that also create an infection risk? I think there should be a sink specifically for families in the intensive care unit. (Participant 3)

Similarly, another parent emphasized the physical challenges of remaining with their child under inadequate environmental conditions:

They tell us that we can stay with our child. But there's nowhere to stay. We have to sit on a chair the whole time. Of course, we're not expecting luxury, but this affects our own fatigue and even our ability to learn caregiving tasks. At least a recliner or armchair would be enough. (Participant 2)

Parents also identified visitation restrictions as one of the barriers limiting family involvement in the care process and continuity of emotional support. Some participants stated that institutional policies allowing only one parent to remain with the child at a time conflicted with FCC principles. One mother described this situation as follows:

If I stay with my child, my spouse cannot see her. I have to leave before he can come in. But because my daughter doesn't want me to leave, her father cannot come in. If there were a little more flexibility about this, maybe truly FCC could be possible. (Participant 6)

Another important issue highlighted by parents was limited timely access to information regarding their child's condition. Although most participants understood that nurses could not always provide detailed medical information, restricted communication practices created uncertainty and anxiety, particularly during emergencies or when physicians were unavailable. One participant expressed this concern as follows:

Of course, I know nurses can't provide medical information. But sometimes we need to hear even a single update so badly. When we ask about our child's condition while handing over milk at the door, they tell us the physician will provide information. I wish there could be a policy change about this too, so that we could receive information whenever we need it. (Participant 11)

Similarly, some parents emphasized the importance of telehealth‐based communication methods for families who were unable to be physically present at the hospital. Participants suggested that remote communication could serve as an effective strategy to reduce parental anxiety and strengthen family involvement in the care process. One parent described this expectation as follows:

We can't travel to the hospital from another city every day. We also have another child at home. When nurses call us about a need and we ask about our child's condition, they say they cannot provide information. We also can't keep calling continuously to get updates. I wish there were an option for families who can't come in to receive information by phone or even see their child through a camera. I think that would be a procedure that could help reassure all families. (Participant 8)

The integration of quantitative and qualitative findings was presented using a joint display approach. As shown in Table 5, quantitative findings were compared with related qualitative themes and parent narratives to identify convergent, complementary, and divergent findings, thereby generating meta‐inferences regarding parents' experiences of family‐centered care in the PICU.

**TABLE 5:** Integration of quantitative and qualitative results (joint display).

Quantitative findings	Related qualitative theme	Example from qualitative findings	Integrative interpretation
The total FCCAS score indicated that parents generally had positive perceptions of FCC: median = 81.0 (Q1–Q3: 73.0–96.3)	Parental perceptions and expectations regarding FCC	Parents associated FCC with receiving high‐quality care, being informed, being able to stay with their child, and participating in care.	Parents' generally positive perceptions were associated with the fulfilment of the core components of FCC. However, the qualitative findings demonstrated that parents defined FCC not only as satisfaction with care, but also as a multidimensional experience involving information sharing, emotional reassurance, physical presence, and participation in care
The support subscale score indicated positive perceptions of support: median = 38.0 (Q1–Q3: 33.0–46.0)	Communication with nurses and perceived support	Parents emphasized nurses' compassionate attitudes, supportive communication, and contributions to teaching caregiving practices	High support scores were directly associated with nurses' empathetic approach and the quality of communication. This finding suggests that nurse–parent communication is one of the key components shaping positive perceptions of FCC
			Although perceived communication was not significantly associated with FCCAS scores, parents consistently described communication as central to feeling supported, informed, and involved in care. This discrepancy suggests that standardized questionnaire scores may not fully capture the complexity of communication experiences in the PICU
The respect subscale score was median = 14.0 (Q1–Q3: 12.0–15.0). Employment status was significantly associated only with the respect subscale; employed parents had higher respect scores than unemployed parents (*U* = 151.00; *p* = 0.039; *r* = 0.28)	Experiences of involvement in the child's care	Some parents reported that their opinions were not always sought and that they felt hesitant to participate in care when they were not explicitly encouraged by nurses	Because the respect subscale showed relatively low internal consistency, this quantitative finding should be interpreted cautiously. Parents reported that they were not sufficiently involved in decision‐making processes and that their individual needs were not always taken into consideration. This finding suggests that supportive communication does not always translate into shared decision‐making, individualized care, or recognition of the parent as an active partner in the care process
The total number of children was significantly associated with the FCCAS total score and the support subscale. Parents with one child had higher total scores than parents with three or more children (*χ* ^2^ = 8.00; *p* = 0.018; *ε* ^2^ = 0.13; post hoc *p* = 0.004; *r* = 0.67). Similarly, support scores were higher among parents with one child (*χ* ^2^ = 8.14; *p* = 0.017; *ε* ^2^ = 0.13; post hoc *p* = 0.007; *r* = 0.64)	Factors facilitating and hindering FCC	Parents with other children reported difficulties in the care process due to responsibilities at home, challenges in visiting the hospital regularly, and family role burdens	The exploratory quantitative pattern related to the number of children was consistent with qualitative narratives emphasizing parents' family caregiving burden. This finding suggests that FCC approaches may need to consider not only the hospitalized child but also the family's overall caregiving burden and responsibilities at home
Parents who reported a high level of participation in the care process had higher FCCAS total scores (*U* = 153.50; *p* = 0.009; *r* = 0.37) and support subscale scores (*U* = 165.00; *p* = 0.018; *r* = 0.34)	Experiences of involvement in the child's care	Parents stated that being involved in simple caregiving practices made them feel more competent, more secure, and closer to their child	Parental participation appeared to be closely associated with FCC perceptions in this exploratory analysis
			Participation functioned not only as a caregiving activity, but also as a mechanism supporting the continuation of the parental role, enhancing feelings of security, and strengthening perceived support
No significant differences were found according to educational status, health literacy, perceptions of communication with healthcare professionals, length of PICU stay, or other non‐significant subscale comparisons (*p* > 0.05)	Factors facilitating and hindering FCC	Parents' experiences varied not only according to demographic characteristics, but also according to nurses' approaches, communication styles, physical environmental conditions, and institutional practices	The absence of significant differences across many sociodemographic variables suggests that perceptions of FCC may be shaped more by relational, organizational, and contextual factors than by individual characteristics. The qualitative findings explained this quantitative result through communication, professional attitudes, the physical environment, and institutional practices

## 6 Discussion

This mixed‐methods study demonstrated that parents generally perceived FCC positively, although these perceptions were not consistently reflected across all FCC dimensions. In particular, although parents perceived a high level of support from healthcare professionals, limitations were identified regarding their participation in decision‐making processes and being regarded as equal partners in care. When the quantitative and qualitative findings were evaluated together, FCC in the PICU appeared to be experienced primarily as a supportive model of care; however, it did not always evolve into a fully partnership‐based care relationship. Overall, integrating both datasets showed that positive FCC perceptions did not necessarily reflect full implementation of all FCC principles, particularly with respect to shared decision‐making and individualized parental involvement. In the present study, the high total scores on the FCCAS suggest that the principles of FCC were adopted to a certain extent in the PICU. Previous studies have shown that FCC practices positively influence parental satisfaction, feelings of trust, care experiences, and psychological adjustment (Stalder et al. 2024; Terp et al. 2021). When interpreted together, the qualitative findings suggested that parents' positive FCC perceptions were largely driven by nurses' compassionate attitudes, supportive communication, and attentiveness toward their children. The qualitative findings helped explain this apparent discrepancy by showing that parents could report positive overall FCC perceptions while simultaneously describing unmet expectations regarding participation and shared decision‐making.

Although total FCC scores were high in the present study, the marked differences observed among the subdimensions indicate that FCC is not a homogeneous construct. Although FCCAS scores suggested generally positive perceptions of FCC, the qualitative findings indicated that some limitations remained regarding shared decision‐making, individualized care, and recognition of parents as active partners. Existing evidence suggests that although parents generally evaluate intensive care experiences positively, deficiencies remain in shared decision‐making, individualized care, and parental autonomy (Dias Lessa et al. 2026; Terp et al. 2021). In the study by Terp et al. the concept of “partnership,” which forms the foundation of FCC, was described by parents as an “unfinished partnership.” In particular, it was emphasized that FCC experiences remained limited when parents were informed but not sufficiently involved in treatment and care decisions (Terp et al. 2021). The qualitative findings of the current study strongly support this interpretation, as some parents reported feeling more like “observers” rather than “active stakeholders” in the care process.

The highest scores observed in the support subdimension indicate that parents perceived strong emotional and informational support, particularly from nurses. In the qualitative findings, parents frequently emphasized experiences such as “not being left alone,” “feeling supported,” “feeling safe,” and “nurses treating their children as if they were their own,” which may help explain this finding. Prior research indicates that an important determinant of parental experience in the PICU is the relational approach of healthcare professionals (Jarvis et al. 2024; Stalder et al. 2024). In a multicenter qualitative study, families identified continuous support, understandable communication, and emotionally accessible healthcare professionals as their greatest needs (Jarvis et al. 2024). Similarly, Stalder et al. reported that parents described nurses' presence as a “balancing,” “reassuring,” and “crisis‐managing” factor during the intensive care process (Stalder et al. 2024). These findings suggest that nurses contribute not only to clinical care delivery but also to parents' psychological sense of safety during PICU hospitalization. Together, these findings indicate that parents' perceptions of support were shaped not only by the availability of care but also by the quality of interpersonal interactions with nurses.

Respect‐related quantitative findings should be interpreted cautiously because the respect subscale showed relatively low internal consistency in the present sample (Cronbach's alpha = 0.55), which may have increased measurement error and reduced the precision of these findings. Therefore, respect‐related results, including the difference according to employment status, should be considered exploratory and interpreted together with the qualitative findings. One of the fundamental principles of FCC, the “shared decision‐making” approach, requires that parents be recognized not merely as informed individuals but as active partners in care. However, existing evidence suggests that due to the highly technological nature of intensive care settings, time pressures, and provider‐centered organizational cultures, parents often remain in passive roles (Charles et al. 2026; Cho et al. 2025). Terp et al. reported that parental participation in care processes largely depended on the attitudes of individual healthcare professionals (Terp et al. 2021). Similarly, the qualitative findings of the current study demonstrated that while some parents were able to participate in care through the supportive attitudes of certain nurses, others encountered professionals whose approaches limited participation. In particular, parents' statements such as “hesitating even to clean my child's face,” “thinking I would not be allowed,” or “feeling that my opinion was not sought” indicate that FCC may still be implemented within provider‐centered structures in practice.

In the exploratory quantitative analysis, participation in the care process was associated with higher FCCAS total and support scores. Although this finding should be interpreted cautiously because of the limited sample size, the qualitative findings suggest that parental participation was an important experiential component of FCC. Taken together, the quantitative and qualitative findings suggest that parental participation was not only associated with higher FCCAS scores but was also experienced as a source of confidence, security, and closeness to the child. Parents described feeling “stronger,” “more valued,” “safer,” and “closer to their child” when they were involved in care. Previous studies similarly demonstrate that parental participation contributes to maintaining the parental role, restoring a sense of control, and reducing stress levels (Boufaied et al. 2026; Poole et al. 2023). In particular, studies examining family‐centered rounds have shown that active parental involvement in the care process increases satisfaction, trust, and collaboration with healthcare professionals (Novianti et al. 2023).

Another notable qualitative finding was that parents associated participation in care with the ability to “maintain the parental role.” In particular, parents of children with chronic illnesses assumed more active roles in the care process, positioned themselves as “the people who know their child best,” and wished to have a voice in care decisions, highlighting the relational dimension of FCC. Prior research emphasizes that parents should be regarded not merely as visitors, but as experiential experts within the child's care team (Boufaied et al. 2026; Terp et al. 2021). From this perspective, the present findings indicate that parental participation in care serves not only a clinical support function, but also a critical role in preserving parental identity.

The largely non‐significant findings regarding demographic variables should be interpreted cautiously given the limited sample size. Nevertheless, when considered together with the qualitative findings, these results suggest that parents' FCC experiences may be shaped more by relational and contextual aspects of care than by individual demographic characteristics. Previous studies similarly indicate that parental satisfaction is associated more with the quality of relationships established with healthcare professionals than with sociodemographic characteristics (Boufaied et al. 2026; Jarvis et al. 2024). These findings suggest that FCC experiences may be shaped more strongly by relational and contextual factors than by individual parent characteristics.

The exploratory finding that parents with one child had higher total FCCAS and support scores than parents with three or more children may offer a preliminary insight into how family caregiving responsibilities relate to parents FCC perceptions. This may be explained by the concentration of parental attention and caregiving energy on a single child. One possible explanation is that healthcare professionals may respond differently to perceived family support needs; however, this interpretation requires further investigation. Indeed, one study emphasized that the degree to which parents are informed about treatment and care was associated with higher healthcare satisfaction and more positive perceptions of FCC (Cetintas et al. 2021). Parents with one child may have been able to devote their full attention to the hospitalization process, enabling them to establish stronger collaboration with healthcare professionals and internalize the support provided more effectively. Conversely, role conflicts and increased caregiving burdens experienced by parents with multiple children due to responsibilities toward children outside the hospital may reduce their perceptions of support received during hospitalization. The qualitative findings helped explain this exploratory association by illustrating how responsibilities toward other children influenced parents' experiences during hospitalization. This finding suggests that FCC implementation should be planned with consideration of holistic family dynamics, including the number of children and caregiving responsibilities at home. Nevertheless, because the number of participants in the one‐child parent group was limited, this finding should be considered exploratory and confirmed in larger samples.

When the variation observed in the quantitative findings is considered together with the diversity of experiences reflected in the qualitative findings, parental experiences appear to be heterogeneous. Although many parents described the care process as supportive and positive, others reported negative experiences related to communication deficiencies, differences in professional attitudes, and inadequacies in the physical environment. Prior studies similarly indicate that FCC implementation may vary across institutions and healthcare professionals (Dias Lessa et al. 2026; Terp et al. 2021). In particular, the finding that parental participation often depended on the approaches of individual professionals suggests that FCC may be shaped more by personal practices than by an institutional culture.

One of the most salient qualitative findings was the influence of physical and organizational conditions on parental experiences. Parents emphasized problems such as the lack of family‐designated sinks, the absence of appropriate resting areas, restrictive visitation policies, and insufficient opportunities to remain beside their child. These findings indicate that FCC is associated not only with professional communication but also with environmental infrastructure. Previous studies demonstrate that family‐friendly physical environments strengthen parental participation in care, psychological safety, and bonding with the child. In particular, Poole et al. reported that one of the most important factors influencing parents' ability to remain at the bedside was the physical and organizational structure of the PICU (Poole et al. 2023). These findings indicate that FCC implementation depends not only on professional behaviours but also on environmental and organizational structures.

Although perceived communication with healthcare professionals was not significantly associated with FCCAS scores in the quantitative analysis, communication emerged as one of the most prominent themes in the qualitative interviews. This discrepancy suggests that standardized questionnaire scores may not fully capture the relational and emotional dimensions of communication experienced by parents in the PICU. Rather than contradicting the quantitative findings, the qualitative findings help explain and expand them by illustrating how communication influences parents' feelings of support, trust, and involvement in ways that may not be fully reflected in overall FCCAS scores. Prior research similarly indicates that open, honest, continuous, and empathetic communication increases parental trust, reduces uncertainty, and facilitates participation in the care process (Boufaied et al. 2026; Jarvis et al. 2024).

The explanatory sequential mixed‐methods design enabled the quantitative findings to be interpreted beyond numerical FCCAS scores. Although parents generally reported positive FCC perceptions and high support scores, the qualitative findings revealed that these positive evaluations coexisted with unmet expectations regarding shared decision‐making, individualized participation, and communication. Likewise, communication was not statistically associated with FCCAS scores, yet parents consistently described it as central to feeling supported, informed, and involved. These findings suggest that standardized questionnaire scores alone may not fully capture the relational and contextual dimensions of FCC in the PICU. By integrating FCCAS scores with parents' narratives, this study provided a more comprehensive understanding of FCC implementation, demonstrating that positive overall perceptions may coexist with important unmet needs that became evident only through qualitative inquiry. Together, the integrated findings highlight aspects of partnership, communication, and individualized parental involvement that would have remained underrecognized if either quantitative or qualitative methods had been used alone.

### 6.1 Limitations

This study has several limitations. First, the quantitative component was conducted in a single PICU with a relatively small sample, which may limit the statistical generalizability of the survey findings to other institutions with different organizational structures, staffing patterns, physical environments, and family‐centered care practices. In addition, because no a priori power analysis was conducted to detect group differences in FCCAS scores, the inferential analyses should be considered exploratory and interpreted cautiously. Second, the qualitative findings are intended to provide an in‐depth understanding of parents' experiences rather than statistical generalizability. To support the transferability of these findings, detailed descriptions of the study setting, participant characteristics, sampling strategy, and data collection and analysis procedures were provided, allowing readers to judge their applicability to other PICU contexts. Third, the study findings were based on parents' self‐reports and interview narratives and may therefore have been influenced by recall or social desirability bias. Fourth, the cross‐sectional quantitative design did not allow changes in parents' perceptions of FCC over time or following interventions to be examined. Fifth, the relatively low internal consistency of the respect subscale limited confidence in respect‐related quantitative findings. Accordingly, interpretations regarding this dimension should be considered exploratory and interpreted alongside the qualitative findings. Finally, multiple subgroup comparisons were performed across the FCCAS total and subscale scores, which may have increased the risk of type I error despite the use of Bonferroni correction for post hoc analyses. Accordingly, statistically significant subgroup findings should be interpreted as exploratory and confirmed in larger, multicenter studies.

## 7 Conclusion

In conclusion, integrating the quantitative and qualitative findings provided a more comprehensive understanding of parents' experiences of family‐centered care in the PICU than either method alone. Although parents generally reported positive perceptions of FCC on the FCCAS, the qualitative findings revealed that these positive perceptions coexisted with unmet expectations regarding participation in decision‐making and individualized care. This suggests that positive FCCAS scores alone may not fully reflect the complexity of parents' experiences or the extent to which all principles of FCC are implemented in clinical practice. Overall, these findings suggest that evaluating FCC solely through standardized questionnaire scores may overlook important dimensions of parents' experiences. Combining quantitative assessment with parents' narratives provides a more comprehensive basis for identifying priorities for clinical practice and for designing interventions that strengthen partnership, shared decision‐making, and family participation in the PICU.

## Author Contributions

**Merve Azak:** conceptualization, methodology, supervision, resources, writing – original draft, writing – review and editing. **Naile Yörür:** conceptualization, data curation, investigation, writing – original draft. **İrem Cafrı:** data curation, investigation, writing – original draft, correspondence. **Nihan Korkmaz:** methodology, formal analysis, visualization, writing – original draft, validation.

## Funding

The authors have nothing to report.

## Disclosure

*Statistical analysis statement*: There are statisticians on the author team: İrem CAFRI (Qualitative) Nihan KORKMAZ (Quantitative). The authors affirm that the methods used in the data analyses were appropriately applied to the study design and research context and that the statistical findings were interpreted correctly. The authors agree to take responsibility for ensuring that the statistical approach used in this study was appropriate and correctly conducted and interpreted as a condition to submit to the Journal.

## Conflicts of Interest

The authors declare no conflicts of interest.

## Data Availability

The research data are not publicly shared. During the ethical approval process, it was formally declared to the Institutional Review Board that the collected data would be used exclusively for the purposes of this specific study. Therefore, in accordance with the approved protocol and the conditions of ethical clearance, the dataset cannot be made publicly available or shared with third parties. This restriction is in place to ensure full compliance with the ethical commitments provided to the ethics committee and to protect participant confidentiality.
